# Assessment of the Diagnostic Performance of a Novel SARS-CoV-2 Antigen Sealing Tube Test Strip (Colloidal Gold) as Point-of-Care Surveillance Test

**DOI:** 10.3390/diagnostics12051279

**Published:** 2022-05-21

**Authors:** Alessandro Medoro, Sergio Davinelli, Serena Voccola, Gaetano Cardinale, Daniela Passarella, Nicola Marziliano, Mariano Intrieri

**Affiliations:** 1 Department of Medicine and Health Sciences “V. Tiberio”, University of Molise, 86100 Campobasso, Italy; alessandro.medoro@unimol.it (A.M.); daniela.passarella@unimol.it (D.P.); nicola.marziliano@unimol.it (N.M.); intrieri@unimol.it (M.I.); 2 Centro Diagnostico Delta S.r.l., Consorzio Sannio Tech, Apollosa, 82030 Benevento, Italy; serena.voccola@tecnobios.com (S.V.); gaetano.cardinale@tecnobios.com (G.C.); 3 Clinical Pathology Laboratory, ASST Rhodense, Rho, 20017 Milan, Italy

**Keywords:** SARS-CoV-2, COVID-19, antigen test, lateral flow assay, nucleocapsid, RT-PCR, clinical validation, nasal swab, nasopharyngeal swab

## Abstract

Severe acute respiratory syndrome coronavirus 2 (SARS-CoV-2) variant outbreaks have highlighted the need of antigen-detecting rapid diagnostic tests (Ag-RDTs) that can be used at the point-of-care (POC). Although many Ag-RDTs have been approved for SARS-CoV-2 detection, studies demonstrating the clinical performance of Ag-RDTs against variants of concern, especially the new Omicron variant, are limited. The aim of this study was to evaluate the diagnostic sensitivity and specificity of the AMAZING COVID-19 Antigen Sealing Tube Test Strip (Colloidal Gold) in 584 early symptomatic and asymptomatic participants (age range 0–90 years). The performance of this Ag-RDT was assessed by comparing its results with reverse transcription RT-PCR (rRT-PCR). One hundred twenty positive samples were also analyzed with rRT-PCR to discriminate Omicron and Delta/Kappa variants (72.50% Omicron; 27.50% Delta/Kappa). Overall, the Ag-RDT showed high positive and negative percent values of 92.52% (95% CI, 86.61–95.95%) and 98.05% (95% CI, 96.41–98.95%), respectively, as well as an overall diagnostic accuracy of 96.92% (95% CI, 95.17–98.16%). Taken together, these data indicate that this inexpensive and simple-to-use Ag-RDT presents excellent analytical performance and can reliably detect Omicron and Delta/Kappa variants.

## 1. Introduction

The global pandemic of severe acute respiratory syndrome coronavirus 2 (SARS-CoV-2) and especially the related in vitro diagnostic tools for rapidly detecting SARS-CoV-2 antigen (and in turn diagnosing early coronavirus disease 2019 (COVID-19)) are having an enormous impact on laboratory medicine. In fact, the managed care of patients with SARS-CoV-2 infection involves early identification, rapid isolation, contact tracing, and establishment of infection prevention and control (IPC) measures [1,2]. Although the diagnostic gold standard remains reverse transcription real time-polymerase chain reaction (rRT-PCR), this assay appears poorly sustainable in screening or surveillance settings since it is expensive, time consuming, and requires special equipment and skilled technicians.

The emergence of variants of concern (VOC) has highlighted an increasing need for rapid and easy to use diagnostics to limit disease transmission [3]. Currently, there is a large number of commercially available antigen-detecting rapid diagnostic tests (Ag-RDTs) that can be used at the point-of-care (POC) [4]. Ag-RDTs are based on direct detection of SARS-CoV-2 viral proteins produced by replicating virus in respiratory secretions, particularly nasal swabs. Most Ag-RDTs allow results in approximately 15–30 min, using anti-SARS-CoV-2 monoclonal antibodies to detect viral antigens in the lateral flow immunoassay format [5]. However, the target of the Ag-RDTs is a crucial aspect of SARS-CoV-2 infection detection by VOC. Three main structural proteins constitute the envelope packing the genome: (1) the membrane (M) protein, a key glycoprotein assembling the viral particles by interacting with all of the other structural proteins; (2) the spike (S) protein, a transmembrane glycoprotein forming homotrimers that protrude from the viral surface and mediate SARS-CoV-2 entry into host cells; (3) the envelope (E) protein, a multifunctional protein, supposed to act on viral assembly, virions release, and pathogenesis. A capsid, surrounding the envelope, is composed by a fourth structural protein, known as the nucleocapsid (N) protein. [6,7,8]. Due to the relative abundance and conserved structure characterized by a low mutation rate, Ag-RDTs were successfully developed and applied for rapid detection of N protein [9,10]. Indeed, the Omicron variant contains only two mutations in the region coding for the N protein compared to more than 30 mutations identified in the S protein [11]. 

Despite this, there is concern regarding the diagnostic sensitivity and specificity of Ag-RDTs, the related reduced diagnostic performance is mainly associated to the relative limit of the target detection [12,13]. The current World Health Organization (WHO) interim guidance recommends that Ag-RDTs meet a minimum of performance requirements of ≥80% sensitivity and ≥97% specificity [14]. A comprehensive meta-analysis has summarized the data of 133 studies evaluating the accuracy of 61 different Ag-RDTs. Across all meta-analyzed samples, the results show a pooled sensitivity and specificity of 71.2% and 98.9%, respectively [15].

However, the timing of detection is also particularly important for VOC, especially Delta (already present in Northern Italy in the early phases of the second pandemic wave) and Omicron variants [16]. The latter variant has shown to have a shorter incubation period than previous VOC, and therefore being potentially more transmissible after infection [17,18]. In this study, the AMAZING COVID-19 Antigen Sealing Tube Test Strip (Colloidal Gold) manufactured by Amazing Biotech Co., Ltd. was evaluated which aims to qualitatively detect the SARS-CoV-2 N protein within the first seven days of symptoms onset, especially for the widely diffused Omicron variant. This test is intended for self-testing at home and can be applied for both nasal and saliva swabs specimens.

## 2. Materials and Methods

### 2.1. Study Design and Population Study

The study was designed to assess the diagnostic performance in SARS-CoV-2 detection of AMAZING COVID-19 Antigen Sealing Tube Test Strip (Colloidal Gold) manufactured by Amazing Biotech Co., Ltd., Xinchang Town, Pudong New Area, Shanghai, China, comparing results of this Ag-RDT with those of the rRT-PCR reference test. It was carried out between December 2021 and February 2022 at the diagnostic laboratory “Centro Diagnostico Delta” in Apollosa, Benevento, Italy, recruiting a total of 584 consecutive patients of any age (range 0–90 years, mean age 40.87). Patients were informed about the ongoing study and, after signing informed consent, were asked to answer a COVID-19 symptom-based screening questionnaire. For minors, parental consent was required. 

As part of the routine diagnostic procedure, the patients enrolled were self-reporting asymptomatic or individuals with mild clinical symptoms such as fever, cough, sore throat, malaise, fatigue, headache, muscle pain, nausea, vomiting, diarrhea, loss of taste and smell (according to the NIH COVID-19 Treatment Guidelines [19]), or asymptomatic individuals reporting close contact to confirmed COVID-19 cases or simple routine screening. Altough the test was developed to detect SARS-CoV-2 N protein within the first seven days of symptoms onset to achieve an early detection, it was possible to enroll only patients showing symptoms for a maximum of three days. The breakdown of duration of symptoms was 1 day in 71 patients, 2 days in 70 patients, and 3 days in 42 patients.

### 2.2. Sample Collection

The nasal and nasopharyngeal swab samples were collected from each patient during daily clinical practice by professional medical staff in close accordance with the sampling methods of AMAZING COVID-19 Antigen Sealing Tube Test Strip (Colloidal Gold) and IVD-validated SARS-CoV-2 Real Time kit (Nuclear Laser Medicine s.r.l, Milan, Italy). Both specimens were collected simultaneously as follows: the sampling swab was inserted into the nasal/nasopharyngeal cavities and rotated gently, making 10 complete circles. After collection, nasal and nasopharyngeal swab samples of each patient were individually put in sterile tubes, blindly numbered and stored at 4 °C until further analysis. Nasal swab samples were processed and tested with the experimental Ag-RDT and nasopharyngeal swabs for RT-PCR reagent detection within 2 h from collection, in accordance with the rules of microbiologic safety, using full personal protective equipment in a laboratory qualified for SARS-CoV-2 detection. 

### 2.3. Principles of Antigen Detection with the AMAZING COVID-19 Antigen Sealing Tube Test Strip (Colloidal Gold)

AMAZING COVID-19 Antigen Sealing Tube Test Strip (Colloidal Gold) is based on a double antibody sandwich method immunochromatographic assay for a highly sensitive detection of COVID-19 N protein in human nasal secretion, providing invisible test area (T) and control area (C). The kit uses colloidal gold to label anti-human coronavirus monoclonal antibody 1; coronavirus monoclonal antibody 2 and polyclonal antibody goat anti-mouse IgG are coated on a nitrocellulose membrane. 

Briefly, nasal swab is processed with the provided extraction buffer and loaded into the strip through which it migrates without any external forces (lateral-flow analysis assay, LFA). In case of positive tested sample, the antigen in the specimen binds first to the antibody 1 labeled with colloidal gold; following its flow to the detection area where it binds to the pre-coated anti-human coronavirus monoclonal antibody 2 to form a double antibody sandwich complex, which generates a colorimetric reaction. Both antibody 1 and antibody 2 bind specifically the SARS-CoV-2 N protein. The remaining colloidal gold-labeled antibody is combined with the polyclonal antibody at the quality control line generating the same colorimetric reaction. Negative samples generate color only at the quality control line level.

If sufficient SARS-CoV-2 antigens are present in the sample, a visible line corresponding to the test area (T) appears. As process control, a colored band must appear in the control area (C) to confirm that sufficient sample has been absorbed from the strip. The test result is visually interpreted after 15 min basing on the presence or absence of visually recognizable colored lines. If the test shows the presence of both C and T bands, the result is positive. The color intensity of the band in the test area (T) may depend on the concentration of SARS-CoV-2 antigens in the sample. Therefore, any colored or shaded band in the test area (T) must be considered as a positive result. The test is negative when there is the only of C band present, while it is invalid when no colored band appears at C zone level, no matter whether the T band appears. In this case, it is necessary to test the sample again with a new tube strip.

### 2.4. Real-Time PCR Detection of SARS-CoV-2 RNA

Molecular detection of SARS-CoV-2 was performed on CFX96 Touch Real Time PCR Detection System (Bio-Rad, Hercules, CA, USA) with the dual probe-based IVD-validated SARS-CoV-2 Real Time kit (Nuclear Laser Medicine s.r.l, Milan, Italy) according to the manufacturer’s instructions, as described elsewhere [20]. Nasopharyngeal swabs were incubated in 350 μL of a provided Extraction Buffer by vigorously vortexing for 1 min, followed by a heat inactivation step at 95 °C for 10 min. Then, samples were briefly spun down and 13 μL of extract with 7 μL of SARS-CoV-2 Mix was used for testing. 

The target genes for SARS-CoV-2 RNA detection with rRT-PCR were the *E* gene, chosen as the target since it is highly conserved among Betacoronavirus genera, and the *RdRp*/*Hel* and *N* genes, that allow the specific discrimination of the SARS-CoV-2 virus. The Internal standard gene is the endogenous *RNaseP* and, therefore, it is extracted together with the sample to monitor the entire procedure (including the integrity of the sample). Two controls are included: a negative control, for both extraction and amplification phases, and a positive control for amplification step only. The internal control includes an internal sequence similar for length and base composition to SARS-CoV-2 target sequence and specific primers and probe to distinguish between internal control and SARS-CoV-2. The positive control is obtained by serial dilutions of a synthetic RNA of cloned *RdRp*, *E* and *N* genes.

According to the manufacturer’s instructions, a sample was positive for SARS-CoV-2 infection when at least one gene between *N* and *RdRp*/*Hel* was specifically amplified at or before the Cycle Threshold (Ct) of 40. 

In addition to the detection of the presence of SARS-CoV-2 in nasopharyngeal swabs, the positive specimens were analyzed to characterize the SARS-CoV-2 lineage according to the GISAID repository (www.gisaid.org (accessed on 5 March 2022)).

In detail, IVD-validated Bosphore^®^ SARS-CoV-2 Variant Detection Kit v4 (Anatolia Geneworks, Sultanbeyli/Instanbul, Turkey), a rRT-PCR method, was used to detect and characterize L452R and E484Q mutations of SARS-CoV-2, which are two of the defining mutations in lineage B.1.617.2 (Delta variant) and B.1.617.1 (Kappa variant). Detection of L452R and E484Q mutations is achieved in a single reaction. The reaction is performed in one PCR tube with the PCR Master Mix. SARS-CoV-2 L452R and E484Q target regions are amplified, and fluorescence detection is accomplished using FAM (L452R) and HEX (E484Q) filters. The fluorescent signal generated by the internal control amplification is detected through Cy5 (*RNaseP*) channel. 

On the other hand, IVD-validated Flame COVID-19 VARIANTS qPCR Master Kit (GVS, Bologna, Italy) was used to identify both circulating forms of the SARS-CoV-2 lineage B.1.1.529 (BA.1 and BA.2), also called Omicron variant, thanks to the positive identification of one of the specific mutations in the *S* gene, the N501Y, shared by the two forms. This kit is designed with two pairs of specific primers and Taqman probes for Omicron variant specific N501Y mutation site. The PCR reaction system contains primers and probes for internal standard. The process of sample collection, extraction and reaction is monitored by detecting internal standard to avoid false negative results.

### 2.5. Statistical Analysis

The performance of the Ag-RDT was evaluated by using Medcalc Software. rRt-PCR was considered the reference method. Accuracy (%), sensitivity (%), positive percent agreement (PPA), negative percent agreement (NPA) and Cohen’s kappa coefficient (κ) [21] were used to determine the diagnostic performance of the Ag-RDT.

## 3. Results

In this study, a total of 584 nasal and nasopharyngeal swab samples were simultaneously collected for clinical performance evaluation by comparing the AMAZING COVID-19 Antigen Sealing Tube Test Strip (Colloidal Gold) and the reference reagent SARS-CoV-2 Real Time Detection kit (Nuclear Laser Medicine S.r.l, Milan, Italy). Samples patients included 277 males (47.43%) and 307 females (52.57%) ranging from 4 months to 90 years of age (Table 1). Different collection sites were used since, as specifically indicated by the manufacturer’s instructions, the AMAZING Ag-RDT is intended to detect SARS-CoV-2 in nasal specimens and not in nasopharyngeal ones, considering that this Ag-RDT is principally intended for self-test screening. 

In detail, 401 (68.7%) participants declared no symptoms, whereas 183 (31.3%) declared at least one symptom among the following ones: fever >37.5 °C; cough; sore throat; malaise; head- ache; muscle pain; nausea; vomiting; diarrhea; loss of taste and smell. 

According to the rRT-PCR, 464 (79.4%) samples were negative and 120 (20.6%) were positive. Among the positive cases, the average cycle threshold (Ct ± standard deviation) values were 27.66 ± 7.05 (min Ct 14; max Ct 45) for the *RdRp*/*Hel* gene and 23.93 ± 4.93 (min Ct 12, max Ct 38) for the *N* gene. 

Overall, the population under investigation showed a positivity rate of 20.6%. Using the rRT-PCR as a reference method, the clinical performance of the Ag-RDT detecting was assessed giving the following results: (1) 111 out of 120 SARS-CoV-2 actively infected patients were confirmed positives and 9 false positives; (2) 455 out of 464 negative patients were confirmed negatives with 9 resulting false negatives (Table 2). PPV and NPV values were respectively 92.52% (95% CI, 86.61–95.95%) and 98.05% (95% CI, 96.41–98.95%) with a diagnostic accuracy of 96.92% (95% CI, 95.17–98.16%) and a general agreement between the rRT-PCR and the Ag-RDT of 90.6% (κ coefficient = 0.906; 95% CI, 0.863–0.984). 

Four of the nine patients who tested false negative were in day-one or day-three of symptoms. Interestingly, in all of these patients *N* gene was amplified, although N protein was not detected by AMAZING COVID-19 Antigen Sealing Tube Test Strip (Colloidal Gold). The average Ct values in false positives were 28.14 ± 9.05 for the *RdRp*/*Hel* gene and 25.15 ± 4.62 for the *N* gene, results not very different from the average values found in the positive population analyzed (Table 3).

The data from Table 2 were also analyzed considering the presence or not and the duration of symptoms at the time of the test. Taking into consideration data coming from asymptomatic patients analyzed with the AMAZING COVID-19 Antigen Sealing Tube Test Strip (Colloidal Gold), 42 patients tested positive and 348 tested negative with 6 false positive and 5 false negative (Table 4). PPV was 93.19% (95% CI, 86.01–96.82%), NPV was 97.27% (95% CI, 93.96–98.79%) and the overall diagnostic accuracy was 96.46% (95% CI, 94.15–98.04%). In the considered asymptomatic population, the agreement between the rRT-PCR and the Ag-RDT was 86.9% (κ coefficient = 0.869; 95% CI, 0.793–0.945).

Considering samples from patients in day-one of symptoms, 31 of them tested positive and 37 tested negative with the AMAZING COVID-19 Antigen Sealing Tube Test Strip (Colloidal Gold); 3 samples gave discordant results compared with rRT-PCR-based reference test with a false positive and 2 false negative (Table 5); PPV was 90.25% (95% CI, 57.20–98.47%), NPV was 98.41% (95% CI, 94.17–99.58%)and the overall diagnostic accuracy was 96.66% (95% CI, 89.42–99.48%). In this subpopulation, the agreement between the rRT-PCR and the Ag-RDT was 91.5% (κ coefficient = 0.915; 95% CI, 0.821–1.000).

Twenty-three samples from patients in day-two of symptoms tested positive and 45 tested negative with the AMAZING COVID-19 Antigen Sealing Tube Test Strip (Colloidal Gold); only 2 samples gave discordant results in which the Ag-RDT resulted negative (2 false negative), and the rRT-PCR tested positive (Table 5). Hence, the PPV and NPV were 85.91% (95% CI, 61.10–95.95%) and 100%, respectively, while the diagnostic accuracy was 96.62% (95%CI, 89.28–99.47%). In this subpopulation, the agreement between the rRT-PCR and the Ag-RDT was 93.7% (κ coefficient = 0.937; 95% CI, 0.850–1.000).

Considering samples from patients in day-three of symptoms, 15 patients tested positive and 25 tested negative with the AMAZING COVID-19 Antigen Sealing Tube Test Strip (Colloidal Gold), while 2 samples (2 false negative) had discordant results compared with rRT-PCR-based reference test (Table 5); PPV was 100%, NPV was 97.04% (95% CI, 89.91–99.18%) and diagnostic accuracy was 97.58% (95% CI, 87.37–99.94%). In this subpopulation, the agreement between the rRT-PCR and the Ag-RDT was 89.9% (κ coefficient = 0.899; 95% CI, 0.764–1.000).

The further objective of this clinical performance study was to discriminate the presence of the Omicron variant related N501Y mutation from the Delta/Kappa variants related L452R and E484Q *S* gene mutations in the 120 positive samples identified in the first phase of the study. In this context it is important to highlight that the N501Y mutation is not Omicron-specific and is also present in other variants [22]. However, the Omicron variant is the dominant strain and the only one with the N501Y mutation currently present in Italy. To strengthen data coming from samples identified positive to Omicron variant, negative samples were tested with Bosphore^®^ SARS-CoV-2 variant Detection Kit to detect the Delta/Kappa variants related L452R and E484Q *S* gene mutations. 

The results coming from the 120 samples analyzed were the following:

- A total of 87 samples (72.50%) were positive to Omicron variant N501Y *S* gene mutation
- A total of 33 samples (27.50%) were positive to Delta/Kappa variants L452R and E484Q *S* gene mutations

Of the 87 samples positive to Omicron variant N501Y *S* gene mutation, 8 samples gave negative results with the AMAZING COVID-19 Antigen Sealing Tube Test Strip (Colloidal Gold), while resulted positive in the RT-PCR reference test results and 79 samples confirmed the positivity in both assays. Hence, the sensitivity of the product AMAZING COVID-19 Antigen Sealing Tube Test Strip (Colloidal Gold) to the Omicron variant N501Y *S* gene mutation is 90.80% (95% CI, 82.68–95.95%). 

Of the 33 samples positive to the Delta/Kappa variants related L452R and E484Q *S* gene mutations, only 1 sample gave negative result with the AMAZING COVID-19 Antigen Sealing Tube Test Strip (Colloidal Gold) and a positive result with the RT-PCR for Delta/Kappa variant L452R and E484Q *S* gene mutations and 32 samples gave positive test results in both assays. Therefore, the sensitivity of the AMAZING COVID-19 Antigen Sealing Tube Test Strip (Colloidal Gold) to the Delta/Kappa variants L452R and E484Q *S* gene mutations is 96.97% (95% CI, 84.24–99.92%).

## 4. Discussion and Conclusions

For more than two years, the global COVID-19 pandemic caused by SARS-CoV-2 has been ongoing. A swift, sensitive, specific, and accurate SARS-CoV-2 detection is still urgently needed in order to limit the epidemic and resurgence of COVID-19 waves caused by new mutations [23]. As a result, LFAs have been playing an essential role in the diagnosis of SARS-CoV-2 infection as a popular and quick diagnostic tool. Many commercial LFA-based Ag-RDTs have been approved for diagnosing COVID-19, and the list keeps growing [24]. On the other hand, despite the availability of several commercial Ag-RDTs for SARS-CoV-2 detection, comparison, and test selection of various commercial Ag-RDTs is still required to screen the most appropriate test. Furthermore, novel LFA strip prototypes with high commercial potential should be based on more extensive research and large-scale clinical validations. Advanced and more sensitive technologies improving diagnostic performance are essential to integrate the actual LFA-based COVID-19 diagnosis kits. Thus, these methods are expected to lay the foundation controlling the COVID-19 pandemic. In this scenario, AMAZING COVID-19 Antigen Sealing Tube Test Strip (Colloidal Gold) has proven to be extremely promising for the following three major reasons [25,26]. Firstly, the target selection of N protein, which is the predominant structural protein of SARS-CoV-2 virus, can be exposed and released in large amounts during virus assembly into the blood, nasopharyngeal aspirate, throat wash samples, saliva, feces, and urine, becoming one of the targets for the early detection of SARS-CoV-2 infection. In this context, detecting N antigen is an effective strategy for the early screening of suspected COVID-19 patients in the early phase of the infection (from asymptomatic, mild symptomatic and symptomatic). Secondly, some people with disability may be particularly anxious about being tested due to the invasiveness of some testing processes. In this context, since AMAZING COVID-19 Antigen Sealing Tube Test Strip (Colloidal Gold) has been validated to analyze also saliva, it can be less uncomfortable than nasopharyngeal swabs for such fragile audience. Thirdly, LFA-based Ag-RDTs have recently drown extensive attention in curbing the spread and resurgence of novel waves of COVID-19 since they can quickly identify new infections by analyzing specific biomarkers of SARS-CoV-2 (nucleic acids, antibodies, and antigens). Indeed, several quick LFA-based diagnostic technologies have been developed and produced as Ag-RDTs playing an important role in controlling COVID-19, particularly in resource-limited situations where RT-PCR assays can’t be performed. Our results prove how the AMAZING COVID-19 Ag-RDT is more successful than the gold standard detection system (rRT-PCR) showing a PPV of 92.52% and a NPV of 98.05%, in line with the FDA requirements.

The AMAZING COVID-19 Antigen Sealing Tube Test Strip (Colloidal Gold) has been developed against the most current SARS-CoV-2 lineages, the Omicron and the Delta/Kappa variants. Although the two lineages detected are discriminated by the S protein and the AMAZING COVID-19 Antigen Sealing Tube Test Strip (Colloidal Gold) targets the N protein, it reports a sensitivity of 90.80% for the Omicron variant and 96.97% for the Delta/Kappa variants. 

The presence of few false-negative cases could be due to inherent analytical variability rather than to the patients’ health status (such as the presence and duration of symptoms at the time of swabbing). However, in all false negative samples, the *N* gene was amplified by rRT-PCR, a result most probably due to the difficulty of the N protein levels detection either due to the potentially very low viral load, as evidenced by some very high *N* gene Ct, or since there is no correlation between the gene and protein expression, in case of low *N* gene Ct values.

Although here we demonstrate the validity of the AMAZING COVID-19 Antigen Sealing Tube Test Strip (Colloidal Gold), we are aware that the report presents the following limitations. Firstly, two different sites of sample collection were used for Ag-RDT and rRT-PCR. The site of sample collection should always be kept as similar as possible in case of test comparison. However, it is necessary to highlight that our sites of sample collection mirror the ones used in real world practice. Secondly, AMAZING COVID-19 Antigen Sealing Tube Test Strip (Colloidal Gold) was not tested for other seasonal viruses (Flu A, Flu B, RSV, etc.) and for previous Coronaviruses (MERF, SARS). Thirdly, the performances of this Ag-RDT toward additional *N* gene mutations—currently addressed by sequencing (conventional Sanger or Next Generation Sequencing) and not by rRT-PCR—were not evaluated. 

In conclusion, AMAZING COVID-19 is a non-laboratory, community-type, household, and POC diagnostic method with the scope of enhancing the community management of COVID-19. To this end, it successfully met its goals. 

## Figures and Tables

**Table 1:** Sex and age distribution of patients enrolled in the consistency comparison test of experimental reagent and reference reagent.

Number of Samples	Patients Sex (N, %)	Mean Patients Age (±SD)
584	Male 277 (47.43%)	40.91 ± 20.32
	Female 307 (52.57%)	

**Table 2:** Overall clinical study results.

AMAZING COVID-19 Test Strip (Colloidal Gold)	PCR Comparator		Subtotal
	Positive	Negative	
Positive	111	9	120
Negative	9	455	464
Subtotal	120 (20.6%)	464 (79.4%)	584

**Table 3:** Presence and duration of symptoms at the time of the test and rRT-PCR results of false negative patients.

Patients	Symptoms	Day from Symptoms Onset	AMAZING COVID-19 Test Strip (Colloidal Gold)	PCR Comparator	Ct
1	No symptoms	-	Negative	Positive	(*N* = 31.29; *E* = 24.03; *RdRp* = 21.09)
2	No symptoms	-	Negative	Positive	(*N* = 18.08; *E* = 19.32; *RdRp* = 16.25)
3	No symptoms	-	Negative	Positive	(*N* = 24.11; *E* = 24.03; *RdRp* = 23.53)
4	Fever >37.5 °C, dry Cough, fatigue, headache, sore throat, diarrhoea	3	Negative	Positive	(*N* = 25.94; *E* = N/A; *RdRp* = 33.28)
5	Dry cough, sore throat	1	Negative	Positive	(*N* = 19.73; *E* = 21.03; *RdRp* = 17.63)
6	No symptoms	-	Negative	Positive	(*N* = 24.04; *E* = N/A; *RdRp* = 32.44)
7	No symptoms	-	Negative	Positive	(*N* = 28.65; *E* = N/A; *RdRp* = 38.20)
8	Dry cough, sore throat	1	Negative	Positive	(*N* = 23.40; *E* = 20.18; *RdRp* = 22.92)
9	Fever > 37.5 °C, dry cough, fatigue, headache, sore throat, diarrhoea	3	Negative	Positive	(*N* = 31.10; *E* = N/A; *RdRp* = 36.25)

**Table 4:** Samples from asymptomatic patients.

AMAZING COVID-19 Test Strip (Colloidal Gold)	PCR Comparator		Subtotal
	Positive	Negative	
Positive	42	6	48
Negative	5	348	353
Subtotal	47	354	401

**Table 5:** Samples from patients in day-one, day-two, and day-three of symptoms.

AMAZING COVID-19 Test Strip (Colloidal Gold)	PCR Comparator		Subtotal
	Positive	Negative	
**Day-two of symptoms**			
Positive	31	1	32
Negative	2	37	39
Subtotal	33	38	71
**Day-two of symptoms**			
Positive	23	2	25
Negative	0	45	45
Subtotal	23	47	70
**Day-three of symptoms**			
Positive	15	0	15
Negative	2	25	27
Subtotal	17	25	42

## Data Availability

Data are available upon reasonable request from the corresponding author.
